# Cognitive Frailty among Older Adults in Rural Areas: Prevalence and Risk Factors

**DOI:** 10.3390/jcm12227019

**Published:** 2023-11-10

**Authors:** Bader A. Alqahtani, Aqeel M. Alenazi

**Affiliations:** Department of Health and Rehabilitation Sciences, Prince Sattam Bin Abdulaziz University, Al-Kharj 11942, Saudi Arabia; aqeelalenazi.pt@gmail.com

**Keywords:** cognitive frailty, MCI, older adults, Saudi elderly, frailty

## Abstract

Background: Cognitive frailty (CF), which is a combination of physical frailty and cognitive impairment, has been associated with functional deterioration in the elderly. However, information about the prevalence of CF and associated factors among Saudi older adults is lacking. Objectives: To assess the prevalence of CF and its associated factors in Saudi community-dwelling older adults. Design: Cross-sectional. Setting: Community-based. Subjects and methods: Thise study included community-dwelling elderly adults aged 60 years and over living in the Riyadh region. This study took place from August 2019 to June 2020. CF was defined as the co-existence of physical frailty and mild cognitive impairment (MCI) without dementia. The association between sociodemographic and clinical factors and CF was estimated using the relative risk ratio and confidence intervals (RRR; CIs 95%) using a multivariable binary logistic regression. Main outcome measures: Fried’s frailty phenotype index; and the Mini-Mental State Examination. Sample size: A total of 421 community-dwelling older adults (63% male; mean [SD] age 70 [7.1] years). Results: The overall prevalence of CF was 6.1%. The following factors were associated with CF: age (RRR 16.3; 95% CI 4.91–54.4), being single (RRR = 3.76 95% CI 1.70–8.31), and number of chronic conditions (RRR 3.1; 95% CI 1.74–5.49). Conclusions: This study indicated the high prevalence of CF among Saudi community-dwelling older individuals compared to other populations. Screening for early diagnosis should be incorporated during examination for older adults. Limitations: The cross-sectional design limits the causality inference with associated risk factors.

## 1. Introduction

The world is confronted with the dilemma of an aging population. There were 703 million people aged 65 and over in the world in 2019 [1]. The population of older adults is expected to increase to 1.5 billion by 2050, which will represent 16% of the population by 2050, implying that one in every six individuals on the planet will be 65 or older [1]. In Saudi Arabia, the elderly population is expected to grow from 5.6 percent in 2017 to 22.9 percent by 2050 [1]. Given the high prevalence of chronic diseases, healthcare systems will face significant challenges as the population of older adults grows dramatically. These chronic conditions and risks, like diabetes [2], arthritis [3], risk of fall [4], and aging-related disorders such as physical frailty and dementia, need careful monitoring and continuous care.

Deterioration in a number of body systems, including musculoskeletal, cardiovascular, sensorimotor, and cognitive skills, has been linked to aging [5]. Frailty has also been linked with older age, which is related to a high risk of falling, susceptibility to decline in health outcomes, limitation in functional status, and later admission to long-term care facilities or nursing homes [6,7]. Frailty and its health implications are more crucial than ever in Saudi Arabia, given the current increase in the population of elderly people [1].

Physical frailty is defined as a clinical syndrome in geriatric patients marked by a greater susceptibility to poor health outcomes [8]. A systematic review found that the Middle East has a pooled prevalence of physical frailty of 39.2% [9]. In addition, more research conducted in Saudi Arabia reported a prevalence of 21% of physical frailty among elderly people [10]. Physical frailty has been shown to be an independent predictor of incidence of falls, deteriorating function, hospitalization, and mortality [11]. This is despite previous data suggesting a connection between frailty and cognitive function [12,13].

Prior research has shown that cognitive impairment is significantly linked to physical frailty in older people, as the two conditions frequently coexist [12,13]. A consensus meeting of members of the International Academy on Nutrition and Ageing (I.A.N.A.) and the International Association of Gerontology and Geriatrics (I.A.G.G.) recently formed the concept of CF. The consensus panel defined CF as the co-existence of physical frailty and mild cognitive impairment (MCI) without dementia. A recent review found that the pooled prevalence of cognitive fragility among community-dwelling older people was 9% [14]. Although the prevalence of CF is considered low in the geriatric population, CF has been related to a higher risk of falling, functional limitations, low quality of life, and high mortality rate [15,16]. CF is considered a reversible condition, which indicates that early assessment and management is important in preventing physical and cognitive function deterioration in community-dwelling older people. Although previous research has reported the prevalence of CF in different populations, no research has been conducted on the prevalence of CF and its related risk factors in community-dwelling older adults in Saudi Arabia. Therefore, the objective of this study was to assess the prevalence of CF in Saudi community-dwelling elderly people.

## 2. Methods

### Study Design, Participants, and Setting

Between August 2019 and June 2020, a cross-sectional design study was conducted on the community primarily in the city of Alkharj in the Riyadh region. In 2020, the city’s overall population was around 425,300 people. Alkharj is one of the Kingdom’s key centers, with significant economic importance and a modern administration. This city is rich in natural resources, has a large geographic region, and has a diverse population. Residents of Al-Kharj city ≥60 years were invited to participate in the current study. We recruited participants mostly through the media, public advertisements, and local residential communities. All eligible participants signed written informed consent forms prior to the start of the study. Approval was obtained from Prince Sattam bin Abdulaziz University’s ethics council in compliance with the Helsinki Declaration’s guidelines. Inclusion criteria included age of 60 years and older as well as able to read and write in Arabic. Exclusion criteria included any unstable or acute medical condition, such as recent fracture, acute stroke, and unstable hypertension, that might impair their capacity to perform the included measurements or if they were not Saudi citizens.

## 3. Measurements

### 3.1. Frailty Measure

We measured physical frailty using Fried’s frailty phenotype index. The overall score was determined using five components: weight loss (self-reported unintended weight reduction of approximately 10 pounds or more in the previous year); having exhaustion (described by participant answers such as “I felt like everything I did was an effort” and “I couldn’t get started” using adapted questions from the Center for Epidemiological Studies Depression (CES-D) scale); slow gait speed (determined by the time it takes to walk 15 feet (approximately 4.57 m), with adjustments for sex and height); and muscle weakness, using hand grip strength on a JAMAR PLUS+^®^ digital handheld dynamometer (Sam-mons Preston, Bolingbrook, IL, USA). The strength data were obtained by two qualified physical therapists, and we used the average of the three measures of the peak force for the dominant hand in kilograms (kg). Throughout the testing, we checked the calibration of the hand dynamometer on a regular basis. We classified handgrip strength data based on sex and body mass index (BMI) and low physical activity using responses from the participant to the Minnesota Leisure Time Activities Questionnaire [8]. A score of 0 or 1 was assigned to each component. Based on their overall score, participants were divided into three groups: robust, with a score of 0; pre-frail, with 1 or 2; and frail, with 3 or more [8]. 

### 3.2. Cognitive Function

The Arabic version of the Mini-Mental State Examination (MMSE) was used to measure cognitive function [17]. Lower MMSE scores indicate impaired cognitive function, whereas higher values suggest normal cognitive function. Participants were categorized into 3 groups: normal cognitive function (MMSE 28–30), mild cognitive function (MMSE 24–27), and severe cognitive impairment (MSSE < 24) [18].

### 3.3. Demographic and Clinical Variables

Gender (male or female), age in years and categorized into 3 groups (i.e., 60–69, 70–79, and >79), marital status (married or other), housing arrangements (including living alone or with others), and educational level (including no formal education, primary school, or middle school and above) were collected as sociodemographic data. A self-report was used to gather information on chronic conditions including diabetes, osteoarthritis, hypertension, cardiovascular disease, depression, and history of falls. Finally, self-reported weight (kg) divided by height (m^2^) was used to calculate BMI. 

### 3.4. Definition of Cognitive Frailty

CF was characterized as having a physical frailty score of ≥3 and an MMSE score of 18 to <24 without dementia. We classified participants with a score between 18 and <24 on the MMSE without physical frailty into the mild cognitive impairment (MCI) group. This cutoff has been used in previous studies [16,19,20]. 

## 4. Data Analysis

Stata 15.1 statistical software was used to examine the data (Stata Corp, 2015, College Station, TX, USA). The mean and standard deviation for continuous sociodemographic data were calculated, whereas percentages were utilized for variables that were categorical. Data normality was assessed using the Kolmogorov-Smirnov test. To compare baseline variables for the four groups (physical frailty, CF, MCI, and robust group), one-way ANOVA or the Kruskal-Wallis test were used, depending on data normality. We used the chi-square test to compare categorical variables. A multinominal logistic regression was conducted to analyze risk factors that were associated with the presence of CF, and the robust group was used as the reference group in the model. The relative risk ratio (RRR) and its associated 95% confidence interval (CI) were reported. Model I provides the crude odds ratio from the univariable logistic regression, while Model II is adjusted for demographic variables, BMI, and number of chronic conditions. The alpha level was determined at <0.05 for statistical significance.

## 5. Results

We included a total of 421 participants in the current study. Table 1 shows the demographics and clinical factors of the participants according to their frailty status. The mean age of the participants was 70 years (SD: 7.1). Approximately 63% (265/421) of the current study sample were men. A total of 21.8% of the participants were classified as having a mild cognitive impairment, 3.3% were physically frail, 6.1% were cognitively frail, and 50% were robust. The prevalence of CF was higher among participants who had low or no formal education and had two or more chronic conditions, as shown in Table 1. 

### 5.1. Differences in Cognitive Function across Frailty Groups 

A one-way ANOVA was conducted to compare cognitive scores across different frailty groups. There was a significant difference in cognitive function, as reported using the MMSE, among frail, pre-frail, and robust participants (*p* < 0.001). The MMSE decreased as the frailty status worsened, with frail individuals having a mean score of 24.7 ± 3.1 (*p* < 0.001 *), pre-frail individuals having a mean score of 26.7 ± 3.2, and robust individuals having a higher mean score of 27.4 ± 2.92 (*p* < 0.001 *) when compared to the pre-frail group. (Table 2). 

### 5.2. Factors Associated with Cognitive Frailty

Age was a significant risk factor associated with CF across all models in multiple logistic regression analyses (Table 3). When compared to individuals aged 60–69 years, the RRR values and 95% CI for older people aged 80 and above increased dramatically to 16.3 (95% CI 4.91–54.4). The adjusted model showed that male gender and number of chronic conditions were significantly associated with CF, with an RRR of 4.31 (95% CI 1.76–10.5) and 3.1(95% CI 1.74–5.49), respectively. Participants who were single (RRR = 3.76 (95% CI 1.70–8.31)) showed a substantially greater risk of CF than those who were married (Table 3).

## 6. Discussion

The prevalence of CF in Saudi older people and associated factor, were investigated in the current study. The prevalence of CF in our study was 6.1%. The present study also found that older age, being single, and number of chronic diseases were all associated with CF. Despite the fact that several studies on the prevalence of CF in other populations have been conducted, no data have been published on CF in Saudi Arabia.

The prevalence of CF in the current study (6.1%) was within the range of a recent meta-analysis reporting the pooled prevalence of CF to be between 6% and 16% [21]. This meta-analysis included 51 studies that used different cognitive measures such as the MMSE, MoCA, and other scales. Due to these differences in frailty assessment and cognitive function assessment, the prevalence of CF was as low as 0.71% and as high as 58% [12,15]. However, the pooled prevalence of CF using the MMSE in this meta-analysis was 6%, which is consistent with our prevalence estimate (6%). This low prevalence estimate could be explained by the Fried criteria of three components or more compared to the other less conservative criteria of one component or more [21]. Another reason was the younger individuals in our study and in other studies with a low prevalence of CF [21].

The risk factors associated with CF in the current study were older age, being single, and number of chronic conditions. These factors have been found to be associated with CF in previous studies [21,22,23]. Older age was consistently a significant risk factor for CF in previous studies [16,23,24]. Aging is a biological change that causes changes at the cellular level, leading to age-related pathological changes including cognitive and physical components [25]. In contrast to previous studies, our study found that being single was associated with CF [23]. These findings could be explained by the cultural differences between countries, such the fact that living with family members is considered the norm in Saudi culture, and the impact of marital status on health and CF. In addition, other factors that might explain such an association include social isolation, potential lifestyle differences, mental health challenges, and the absence of built-in support networks that partnerships or marriages often provide [26,27]. Nonetheless, this association highlights the critical need to address potential risk factors for cognitive frailty among single individuals and promote cognitive health across diverse social contexts. Future studies should explore the relationship between CF, marital status, and psychological factors. An increase in the number of comorbidities was significantly associated with CF in our report. In contrast, a previous work found no association between number of comorbidities and CF [23]. Future research should investigate the possible mechanism between number of chronic conditions and CF.

Clinical implications for the current study include prevention, screening, and intervening for Saudi community-dwelling older adults. Highlighting modifiable associated risk factors such as number of comorbidities is of importance to manage diseases and improve general strength. The management of chronic diseases such as diabetes and hypertension includes medication adherence and lifestyle change involving diet and exercise. Future research should examine whether improvements in these modifiable risk factors lead to improvement in CF.

Future research should take into account these limitations of this study. The cross-sectional design limits the causality inference with associated risk factors. Future work should examine these risk factors at baseline and at follow up. However, this study was the first to report CF among older adults living in Saudi Arabia. Cognitive function was measured using the MMSE, and this is not an objective measure. However, the MMSE has been shown to be a relevant and valid measure for MCI. To assess physical activity, we used a consensus Arabic translation of the Minnesota Leisure Time Activities Questionnaire, which allowed us to collect data effectively. However, there is a need for potential future validation efforts to further establish the questionnaire’s reliability and validity in the Arabic-speaking context. Finally, convenience sampling is another limitation that might affect the generalizability of the results. In addition, our sample was not drawn randomly from the population. Having an unbalanced gender-based sample might contribute to the high prevalence of men in the CF and MCI groups. Future work should consider random sampling to generalize the findings.

This study provided the first prevalence rate in Saudi community-dwelling older adults and examined the associated risk factors of CF. The prevalence of CF in this study was 6.1%. This study suggests that early screening and intervention might be incorporated during examination for older adults to enable early diagnosis. Future studies should examine the longitudinal impact of associated risk factors on CF in this population.

## Figures and Tables

**Table 1:** Demographics and clinical factors according to frailty status groups.

Variable	Total Sample N = 421 (%)	Frailty Status, n (%)					* p *
		Robust (211)	Cognitive Frailty (26)	Physical Frailty (14)	Dementia (78)	MCI (92)	
Age, mean (SD)	70.2 (7.1)	67.8 (5.92)	77 (7.56)	73.1 (7.7)	72.2 (6.8)	71.9 (7.13)	<0.001
Gender ^a^							
Male	265 (62.9)	117 (44.1)	19 (7.1)	8 (3.1)	62 (23.3)	59 (22.2)	0.004
Female	156 (37.1)	94 (60.2)	7 (4.4)	6 (3.8)	16 (10.2)	33 (21.2)	
Education ^a^							
No formal education	256 (60.8)	117 (45.7)	23 (8.9)	8 (3.1)	49 (19.1)	59 (23.1)	<0.001
Primary school	105 (24.9)	80 (76.1)	2 (1.9)	3(2.8)	5 (4.7)	15 (14.2)	
Middle school or more	60 (14.2)	15 (25)	1 (1.66)	3 (5)	23 (38.3)	18 (30)	
Marital status ^a^							
Married	276 (65.6)	165 (59.7)	11 (3.9)	11 (3.9)	41 (14.8)	48 (17.3)	<0.001
Unmarried	145 (34.4)	46 (31.7)	15 (10.3)	3 (2.1)	37 (25.5)	44 (30.3)	
Living arrangement ^a^							
Living with others	368 (87.4)	194 (52.7)	20 (5.4)	14 (3.8)	66 (17.9)	74 (20.1)	0.04
Living alone	53 (12.5)	17 (32)	6 (11.3)	0	12 (22.6)	18 (33.9)	
Number of comorbidities ^b^	408 (96.9)	1.10 (0.87)	1.71 (0.62)	1.70 (0.75)	1.45 (0.77)	1.36 (0.76)	<0.001
BMI (Kg/m^2^), mean (SD) ^c^	421	26.7 (4.5)	24.2 (5.1)	22.5 (3.1)	25.4 (5.4)	27.9 (4.8)	<0.001
MMSE (score), mean (SD)^c^	421	29.2 (0.78)	25.9 (0.95)	28.8 (0.87)	21.3 (1.26)	25.6 (1.1)	<0.001

MCI: mild cognitive impairment; BMI: body mass index; MMSE: Mini-Mental State Examination; SD: standard deviation; ^a^: chi-square test; ^b^: one-way ANOVA; ^c^: Kruskal-Wallis rank test.

**Table 2:** Differences in the cognitive function across physical frailty groups.

Frailty	MMSE		
	Mean ± SD		* p *
Frailty status			
Frail	24.7 ± 3.1		<0.001 *
Pre-frail	26.7 ± 3.2		
Robust	27.4 ± 2.92		<0.001 *

SD: standard deviation; MMSE: Mini Mental State Examination; *: Kruskal-Wallis rank test.

**Table 3:** Factors associated with cognitive frailty.

Independent Variables	Model I	Model II
	RRR (95% CI)	RRR (95% CI)
Age		
70–79	**4.38 (1.90–10.1)**	**3.99 (1.62–9.86)**
≥80	**13.9 (4.88–39.6)**	**16.3 (4.91–54.4)**
Sex (male)	**2.85 (1.30–6.26)**	**4.31 (1.76–10.5)**
Marital status (single)	**3.45 (1.72–6.89)**	**3.76 (1.70–8.31)**
Number of chronic conditions	**2.57 (1.51–4.35)**	**3.1 (1.74–5.49)**
BMI	**0.89 (0.83–0.96)**	**0.88 (0.81–0.96)**

Model I: unadjusted; Model II: adjusted for age, gender, marital status, BMI, and comorbidities. CI: confidence interval; bold font indicate statistical significance (*p* ≤ 0.05).

## Data Availability

Data used in the study are available from the corresponding author on reasonable request.
